# No SOS Needed: A Second Bacterial Checkpoint System Stops Cell Division

**DOI:** 10.1371/journal.pbio.1001978

**Published:** 2014-10-28

**Authors:** Richard Robinson

**Affiliations:** Freelance Science Writer, Sherborn, Massachusetts, United States of America

Cell division must be tightly coordinated with DNA replication so that a complete genome ends up in each daughter cell. That coordination is especially critical when DNA is damaged, as lengthy delays may be required to repair broken strands. To prevent premature division, cells detect the presence of broken DNA with dedicated sensor proteins, which inhibit division while they promote repair.

In bacteria, this “checkpoint” system was originally thought to be the sole responsibility of the so-called SOS response, in which damaged DNA leads to the cleavage of a transcriptional repressor, causing synthesis of both repair enzymes and a cell division inhibitor. However, multiple experiments have shown that even when the SOS response is disabled, some bacteria are able to sense damage and delay division. In this issue of *PLOS Biology*, Joshua Modell, Michael Laub, and colleagues show that a second, independent system works alongside the SOS system, one that is sensitive to a specific type of DNA damage and that induces its own suite of response proteins.

The authors began their search for the mystery inhibitor by inducing DNA damage in cells of the model bacterium *Caulobacter crescentus* in which the SOS system had been inactivated. They found six genes whose up-regulation could not be explained by the actions of known SOS regulators. Through a series of experiments, one gene emerged that bore all the characteristics of a cell division inhibitor and consequently was christened damage-induced cell division inhibitor A, or *didA*.

**Figure 1:**
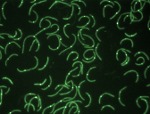
*Caulobacter* cells producing DidA cannot divide as DidA localizes to and blocks the activity of cell division proteins at midcell (DidA-YFP with cell boundaries outlined in white).

Overexpression of the *didA* gene strongly inhibited division (Figure 1) in the absence of SOS proteins, indicating its independence from that system. Conversely, treatment with a DNA toxin in the absence of both *didA* and the SOS system's inhibitor *sidA* led most cells to divide despite the DNA damage, reducing viability.

The presence of both SOS and non-SOS systems introduces redundancy, surely a benefit in such a critical cellular control response. However, the two systems were not responsive to precisely the same insults, the authors found. Both systems responded to a toxin that formed crosslinks between the two strands. The SOS system, but not the *didA* system, was especially responsive to depletion of the nucleotide pool, while the *didA* system, but not the SOS system, responded strongly to creation of double-strand breaks.

A key step in the *Escherichia coli* SOS system is the inhibition of polymerization of the structural protein, FtsZ, which forms a ring at the site of constriction, which is critical for positioning of the division machinery (the “divisome”). In contrast to many bacterial division inhibitors, the authors showed that DidA did not interact with FtsZ. Instead, they found evidence that it most likely binds to a late-arriving member of the divisome, FtsN. However, this interaction, they showed, did not disrupt the divisome or prevent the localization of other members of the complex.

The actual mechanism, they suggest, involves a complex formed among three divisome proteins: FtsN, FtsI, and FtsW. Excess production of DidA normally would shut down cell division, but this effect could be overcome by mutations in either the *ftsW* or *ftsI* genes, despite the fact that DidA bound to neither protein directly. Instead, the authors propose that, in the absence of DidA, the three proteins form an active complex that promotes constriction, and when DidA binds FtsN, it converts the complex into an inactive state.

Finally, the authors showed that expression of *didA* was driven by the transcriptional regulator DriD. Treatment with zeocin, a *didA*-specific DNA toxin, caused DriD to bind to *didA*'s promoter and increase transcription. Zeocin did not, however, up-regulate the *driD* gene itself, indicating that damage-induced posttranslational modification of preexisting DriD protein is a key step in the regulatory pathway.

Together, these results identify a novel mechanism of cell division control in *Caulobacter*. While details will probably differ in other types of bacteria, the identification of a second control system is likely to lead to the search for similar systems elsewhere. In addition, since even disabling both SOS and non-SOS systems did not entirely prevent normal regulation of cell division in *Caulobacter*, the authors note, yet more control systems may remain to be discovered.

**Modell JW, Kambara TK, Perchuk BS, Laub MT (2014) A DNA Damage-Induced, SOS-Independent Checkpoint Regulates Cell Division in** ***Caulobacter crescentus*** **.** doi:10.1371/journal.pbio.1001977

